# Should all hip and knee prosthetic joints be aspirated prior to revision surgery?

**DOI:** 10.1007/s00402-021-03791-6

**Published:** 2021-02-09

**Authors:** Femke Staphorst, Paul C. Jutte, Alexander L. Boerboom, Greetje A. Kampinga, Joris J. W. Ploegmakers, Marjan Wouthuyzen-Bakker

**Affiliations:** 1 Department of Medical Microbiology and Infection Prevention, University of Groningen, University Medical Center Groningen, PO Box 30.001, 9700 RB Groningen, The Netherlands; 2 Department of Orthopaedic Surgery, University of Groningen, University Medical Center Groningen, Groningen, The Netherlands

**Keywords:** Periprosthetic joint infection, Revision surgery, Aspiration, Aseptic loosening, Mechanical failure

## Abstract

**Aims:**

It is essential to exclude a periprosthetic joint infection (PJI) prior to revision surgery. It is recommended to routinely aspirate the joint before surgery. However, this may not be necessary in a subgroup of patients. The aim of our study was to investigate if specific clinical and implant characteristics could be identified to rule out a PJI prior to revision surgery.

**Methods:**

We retrospectively evaluated clinical and implant characteristics of patients who underwent a hip or knee revision surgery between October 2015 and October 2018. Patients were diagnosed with a PJI according to the MSIS diagnostic criteria.

**Results:**

A total of 156 patients were analyzed, including 107 implants that were revised because of prosthetic loosening and 49 because of mechanical failure (i.e. instability, malalignment or malpositioning). No PJI was diagnosed in the group with mechanical failure. In the prosthetic loosening group, 20 of 107 were diagnosed with a PJI (19%). Although there was a significantly lower chance of having a PJI with an implant age of > 5 years combined with a CRP < 5 mg/L, an infection was still present in 3 out of 39 cases (8%).

**Conclusion:**

Implants with solely mechanical failure without signs of loosening and low inflammatory parameters probably do not require a synovial fluid aspiration. These results need to be confirmed in a larger cohort of patients. In case of prosthetic loosening, all joints need to be aspirated before surgery as no specific characteristic could be identified to rule out an infection.

## Introduction

Total joint arthroplasty is one of the most commonly performed surgical procedures in orthopedics. Unfortunately, a prosthetic joint eventually requires revision, either due to mechanical failure (i.e. instability, malalignment or malpositioning) or loosening of the prosthesis [1, 2]. Published records describe that around 10–20% of patients undergoing revision surgery have unexpected positive cultures during revision [3–6], and these patients have a worse outcome compared with aseptic cases [7–10]. Therefore, it is critical to rule out a periprosthetic joint infection (PJI) prior to revision surgery because the revision of an infected prosthesis requires other surgical and antibiotic interventions than a revision for a non-infected prosthesis. Currently, there is no single test to confirm or exclude a PJI. Low serum inflammatory parameters such as C-reactive protein (CRP) and erythrocyte sedimentation rates (ESR) are false negative in a proportion of patients with a low-grade infection [11–14]. For this reason, it is advised not to rely on serum inflammatory parameters to rule out an infection [15]. Consequently, many experts recommend routine synovial fluid analysis prior to revision surgery. In particular, a synovial leucocyte count below 1500 cells/μL and/or negative biomarkers (e.g. alpha defensin or calprotectin) have a very high negative predictive value and may be used in this regard [16–19]. However, it is known that specific clinical and implant characteristics increase the risk of having a PJI, such as revision prostheses and early prosthetic loosenings [20, 21]. On the other hand, prostheses being revised for mechanical reasons may have a lower odd of having a PJI. Along these lines, identifying a subgroup of patients undergoing revision surgery in whom a synovial fluid aspiration is not necessary would be favorable. Therefore, the aim of our study was to investigate if specific clinical and implant characteristics could be identified to rule out a PJI and in whom a synovial fluid aspiration would be redundant.

## Materials and methods

### Study design and inclusion criteria

We performed a retrospective, observational cohort study in which the data of all patients who underwent a one or two-stage revision of a total joint prosthesis were collected and evaluated (October 2015 until October 2018). The following inclusion criteria were applied:

1. Patients undergoing total revision surgery of a hip or knee prosthesis.
2. The revision surgery was performed more than 3 months after initial replacement.

Exclusion criteria:

1. Patients < 18 years of age.
2. Patients with a fracture as an indication for revision.
3. Patients with missing intra-operative tissue cultures.
4. Patients who underwent revision surgery for acute PJI.

Patients were classified as having a PJI according to the Musculoskeletal Infection Society (MSIS) published in 2013 [22]. The indication for revision surgery was divided into two categories: prosthetic loosening or mechanical failure (i.e. instability, malalignment or malpositioning). The patient was assigned to the category prosthetic loosening when there was mechanical failure simultaneously with implant loosening diagnosed on X-ray and/or nuclear imaging (i.e. three-phase bone scintigraphy using ^99m^technetium-hydroxymethylene diphosponate). The study was approved by the local Medical Ethical Committee (Reference Number: 201900356).

### Data collection

The following clinical variables were collected: patients' medical history (including the presence of rheumatoid arthritis, the use of immunosuppressive drugs and/or antibiotics), specific characteristics of the prosthesis (cemented or uncemented, revised or primary, age of the prosthesis at the time of revision, etc.), presence of other osteosynthesis material (OSM) around the prosthesis (e.g. plates or screws), preoperative serum inflammatory markers (CRP and ESR) and details on intra-operative cultures.

### Handling of cultures

During revision surgery, five intra-operative biopsies and one synovial fluid aspiration were obtained for culture. In addition, the prosthesis was vortexed for 30 s in Ringer lactate, sonicated for 1 min at 40.000 Hz, and again vortexed for 30 s. Subsequently, one hundred microliters of sonication fluid was plated on blood agar plates and 10 mL of sonication fluid was incubated in blood culture bottles (BD BACTEC^™^). Each sample and positive sonication fluid was cultured for 9–11 days on blood and chocolate agar under aerobic conditions (with 5% CO_2_) and on Brucella blood agar under anaerobic conditions. In addition, all samples were cultured in fastidious broth. All broths were subcultured on blood and Brucella blood agar after 7 days of incubation. Subcultures were incubated for 2 days. The bacterial species was determined using the matrix-assisted laser desorption/ionization time-of-flight analyzer (MALDI-TOF).

### Statistical analysis

All statistical analyses were performed using IBM SPSS statistic version 23^®^. A Chi-square test (or a Fisher exact test when needed) was used to analyze the difference between the groups for categorical variables, and a one-way ANOVA (or Kruskal–Wallis test when data were not normally distributed) for continuous variables. To analyze the effect of possible risk factors for PJI, a logistic regression analysis was performed. Variables with a significance level of < 0.1 were subsequently analyzed in a multivariate logistic regression model. The Kaplan–Meier curve was used to evaluate the event-free survival time (time between implantation and revision) for three different groups: aseptic loosening, PJI, and mechanical failure. A significance level of 0.05 (*a* = 5%; bilateral) was considered for all analyses.

## Results

### Incidence of PJI in cases revised for mechanical failure versus prosthetic loosening

A total of 156 patients that underwent revision of a hip or knee prosthesis were included (Table 1). None of the patients were on antibiotic treatment at the time from revision. From the 156 patients, 107 had the preoperative diagnosis of prosthetic loosening (69%), while 49 patients were diagnosed solely with mechanical failure (31%). The majority of the prostheses revised for mechanical failure were knees (44 of 49, 90%). Loosened prostheses were revised to the same extent in both the joints (56 hips, 51 knees). Overall, 13% (20 of 156) of patients were diagnosed with a PJI. All 20 PJIs were diagnosed in patients with loosening of the implant, which entailed 19% (20 of 107) of the cohort of patients with prosthetic loosening. No PJIs were diagnosed in the group of patients with mechanical failure. 92% of patients that underwent revision surgery due to mechanical failure had a CRP < 10 mg/L.

**Table 1:** Patient characteristics

	Prosthetic loosening *n* = 107	Mechanical failure *n* = 49	*P* value
Baseline characteristics			
Male sex	47 (44)	19 (39)	0.603
Age, years	67.5 (11.8)	64.6 (11.6)	0.152
Body mass index (kg/m^2^)	28.6 (6.0)	29.6 (5.2)	0.299
Smoking	21 (20)	10 (20)	1.000
Comorbidity			
Hypertension	47 (44)	20 (41)	0.731
Ischemic heart disease	13 (12)	9 (18)	0.327
Heart failure	5 (5)	1 (2)	0.666
Diabetes mellitus	11 (10)	6 (12)	0.784
Chronic obstructive pulmonary disease	8 (8)	4 (8)	1.000
Chronic renal insufficiency	3 (3)	1 (2)	1.000
Liver cirrhosis	1 (1)	1 (2)	0.540
Malignancy^b^	5 (5)	0 (0)	0.177
Malignancy in bone^b^	2 (2)	0 (0)	1.000
Rheumatoid arthritis	9 (9)	2 (4)	0.504
Gout	1 (1)	1 (2)	0.531
Any previous surgery of the affected joint apart from primary placement	60 (56)	23 (47)	0.305
Previous infection joint	15 (14)	2 (4)	0.095
Immunosuppressive medication	9 (8)	3 (6)	0.754
Prosthesis			
Joint			
Hip	56 (52)	5 (10)	0.000^a^
Knee	51 (48)	44 (90)	0.000^a^
Cemented prosthesis	53 (68)	28 (70)	1.000
Type of prosthesis			
Revision prosthesis	36 (34)	6 (12)	0.006^a^
OSM around prosthesis^c^	15 (14)	3 (6)	0.184
PJI	20 (19)	0 (0)	0.000^a^

Nominal variables are depicted as *n* (%), continuous variables are depicted as mean (SD) or median (interquartile range) when not normally distributed

^a^*P* values < 0.05 were considered statistically significant

^b^Active malignancy at the time of revision

^c^*OSM* osteosynthesis material around the prosthesis (e.g. screws, plates)

### Incidence of PJI according to clinical and implant characteristics

**Fig. 1:**
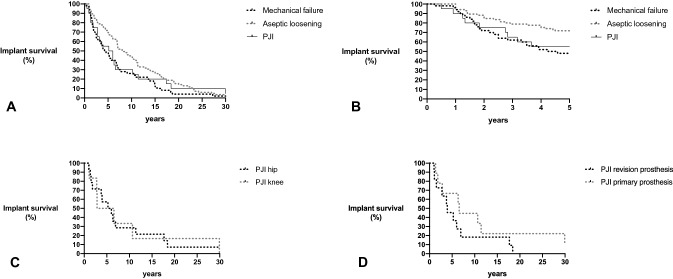
Prosthesis age and implant survival. Kaplan–Meier depicting implant survival (time until revision) due to mechanical failure (*n* = 49), aseptic loosening (*n* = 87) or PJI (*n* = 20), depicted for 30 years (**a**) and 5 years (**b**), respectively, and implant survival in PJI cases according to the type of joint (**c**) and the type of prosthesis (**d**)

To identify which implant characteristics are associated with a PJI, we first analyzed to what extent the age of the prosthesis was associated with the likelihood of having a PJI. Cases diagnosed with a PJI had a similar time to revision as the cases revised for a mechanical reason [mean time to revision 7.9 years (95% CI 3.90–11.90) versus 6.5 years (95% CI 4.61–8.43), respectively, *P* = 0.541 (Fig. 1a, b)], but aseptic loosenings had a considerably longer time to revision [9.8 years, (95% CI 8.08–11.43)]. These cases had the longest survival in the first 5 years with only 32% of cases (28 of 107) needing a revision within this time period. No differences in time to revision were observed in the PJI cases according to the type of joint or the type of prosthesis (Fig. 1c, d).

We additionally investigated if specific implant characteristics could differentiate between septic and aseptic loosening (Table 2). A previously treated infection of the affected joint and/or having a revision prosthesis were both significant risk factors for having a PJI in the univariate analysis, with an unadjusted OR of 5.32 (95% CI 1.65–17.16, *P* = 0.005) and 3.03 (95% CI 1.12–8.21, *P* = 0.029), respectively. When testing for collinearity, 12 of the 36 patients with a revision prosthesis have had a previous infection of the affected joint. A hip prosthesis and/or having a prosthesis age of ≤ 5 years were also associated with a higher odds of having a PJI, but these differences were not statistically significant. None of the analyzed variables were independent predictors for a PJI in the multivariate analysis (Table 2).

**Table 2:** Univariate and multivariate analyses for PJI in patients with loosening of the implant

	Septic loosening (*n* = 20)	Aseptic loosening (*n* = 87)	Unadjusted OR (95% CI)	*P* value	Adjusted OR (95% CI)	*P* value
Implant characteristics						
Previous surgery affected joint	15 (75)	45 (52)	2.80 (0.94–8.38)	0.066*	2.14 (0.47–9.71)	0.327
Previous infection affected joint	7 (35)	8 (9)	5.32 (1.65–17.16)	0.005*	2.82 (0.74–10.75)	0.129
Hip	14 (70)	42 (48)	2.50 (0.88–7.09)	0.086*	2.70 (0.85–8.54)	0.091
Cemented prosthesis	13 (81)	40 (65)	2.38 (0.61–9.28)	0.210		
Revision prosthesis	11 (55)	25 (29)	3.03 (1.12–8.21)	0.029^*^	1.21 (0.29–5.08)	0.794
OSM around prosthesis^a^	4 (20)	11 (13)	1.71 (0.48–6.04)	0.409		
Age of the prosthesis						
≤ 1 year	2 (10)	2 (2)	4.72 (0.62–35.77)	0.133		
≤ 2 years	5 (25)	11(13)	2.30 (0.70–7.60)	0.171		
≤ 3 years	7 (35)	17 (20)	2.22 (0.77–6.40)	0.141		
≤ 4 years	9 (45)	21 (24)	2.57 (0.94–7.05)	0.066*	2.07 (0.68–6.29)	0.197
≤ 5 years	9 (45)	28 (32)	1.72 (0.64–4.64)	0.280		
≤ 10 years	14(70)	51 (59)	1.65 (0.58–4.69)	0.350		
≤ 15 years	16 (80)	62 (71)	1.61 (0.49–5.30)	0.431		
≤ 20 years	18 (90)	75 (86)	1.44 (0.30–7.01)	0.652		

Nominal variables are depicted as *n* (%), continuous variables are depicted as mean (SD) or median (interquartile range) when not normally distributed

*Variables included in the multivariate binary logistic regression analysis

^a^*OSM* osteosynthesis material (e.g. screws or plates)

Patient characteristics (age, sex, smoking status, BMI, comorbidities and use of immune-suppressive drugs) were not associated with having a PJI (data not shown).

### Incidence of PJI in loosened implants and serum inflammatory markers

We additionally analyzed whether a CRP < 5 mg/L and/or an ESR < 30 mm/h could rule out a PJI in patients with prosthetic loosening. Of the 20 patients with septic loosening, 3 patients had a CRP < 5 mg/L (15%). All of these three patients also had an ESR of < 30 mm/h. The causative microorganisms in these three cases were *Staphylococcus epidermidis*, *Corynebacterium amycolatum* and *Staphylococcus lugdunensis*, cultured in three, four and six intraoperative tissue samples, respectively. The patient with the *S. lugdunensis* infection had a sinus tract, which was probably the reason for the low CRP in this patient.

**Fig. 2:**
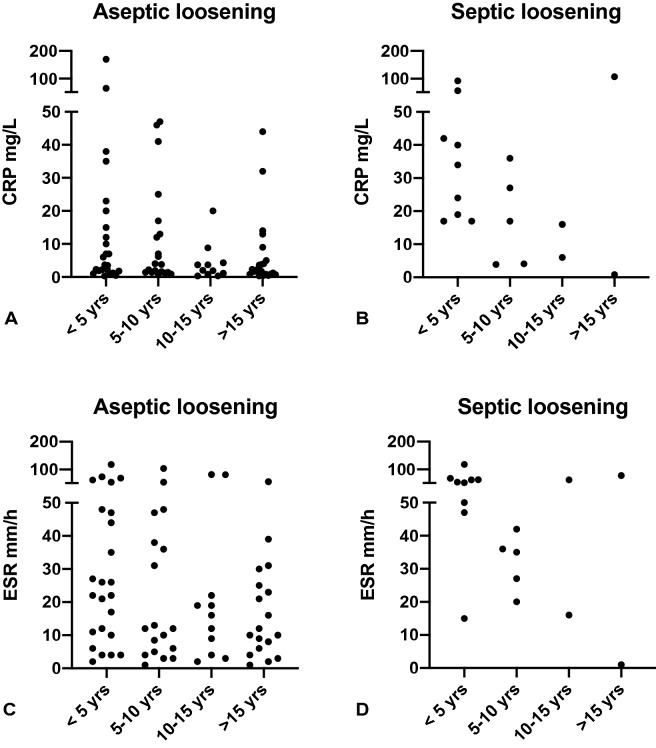
Correlation between serum inflammatory parameters and prosthesis age at the time of revision. Scatterplot C-reactive protein (CRP) and erythrocyte sedimentation rate (ESR) of 76 patients with aseptic loosening (**a**, **c**) and 18 patients with septic loosening (**b**, **d**) (missing data in 11 and 2 and cases, respectively)

Figure 2 shows the serum CRP and ESR levels in relation to the age of the prosthesis at the time of revision. As depicted, a low serum CRP and/or ESR level could not rule out a PJI, even if the prosthesis was > 5 years of age at the time of revision. The incidence of PJI in this group was 8% (3 out of 39). When excluding the patient with the sinus tract, the incidence was 5%.

## Discussion

In this retrospective observational cohort study, we analyzed whether specific clinical or implant characteristics could be identified to rule out a PJI prior to revision surgery without the need for synovial fluid aspiration. Our results indicate that patients who undergo revision surgery due to mechanical failure of the prosthesis probably do not need to be aspirated if accompanied with low-inflammatory parameters. We could not identify a subgroup of patients with loosening of the prosthesis in whom not aspirating the joint would be justifiable.

To our knowledge, we are the first who explicitly report on the incidence of PJI in prostheses with mechanical failure. A study by Khalid et al. did determine the incidence of unrecognized PJI in 72 patients undergoing revision for aseptic failure without any clinical suspicion of infection. These so-called aseptic failures were defined as the patients with implant instability and/or polyethylene wear but also included cases with implant loosening. Within this population, five patients turned out to have an unrecognized PJI (6.9%), but it was not reported if the infections were only observed in the patients with prosthetic loosening [23]. The absence of infection in our patient population with mechanical failure should be interpreted with some caution. It should be noted that the gross majority of patients with mechanical failure in our analysis had low serum inflammatory markers. For this reason, we cannot conclude whether a synovial fluid aspiration is still needed in patients with an elevated CRP. In addition, our results need to be confirmed in a larger cohort of patients with merely mechanical failure, and a subanalysis on hips and knees should be performed. In our study, the majority of patients had mechanical failure of knees. As the aspiration of a hip joint is more complicated compared with knees, this group of patients is of particular interest.

Regarding patients with prosthetic loosening, the incidence of PJI in this particular group was high. The PJI incidence of 19% in patients with prosthetic loosening in our study is comparable with other reports [3–6]. We could not identify a subgroup of patients with prosthetic loosening in whom a PJI could be ruled out preoperatively without synovial fluid aspiration. Commonly described patient-related risk factors for a PJI such as male sex, diabetes, rheumatoid arthritis and obesity did not appear to be risk factors in our study [24–26]. A possible explanation might be that the importance of host-related factors are more pronounced when analyzing the risk of acute PJIs, rather than chronic ones. We did found that certain implant characteristics were associated with a higher odds of having a PJI. In accordance with previous studies [20, 21, 27], early loosening occurred more often in the presence of a PJI. Portillo et al. demonstrated that in the first 2 years after implantation, revisions were performed more often for PJI (69%) than for aseptic loosening (16%). Indeed, in most studies, early loosening is defined as loosening within 2 years after implantation [20, 21]. In our study, the most prominent difference was observed using a cut-off of 4 years instead of 2 years. In addition, we identified that a previously treated infection of the affected joint and/or having a revision prosthesis are both associated with a higher odds of having a PJI. Despite the identification of these implant-related risk factors, PJIs were still detected in our population far after the 4-year implantation period. Moreover, even combined with a low serum CRP, a PJI could not be ruled out, not even when using a lower threshold of 5 mg/L instead of the accepted 10 mg/L used in the diagnostic criteria for PJI [22]. This finding again underlines that we cannot solely rely on serum CRP and ESR, as bacteria are able to remain in a ‘dormant’ and asymptomatic state within a biofilm without inducing any systemic inflammatory response [28, 29].

An important limitation of our study is the limited number of patients with a PJI (*n* = 20), which hampers the possibility to perform detailed subanalyses on high and low-risk groups for infection. Second, the preoperative diagnosis of loosening is challenging. In our cohort, next to conventional X-rays to detect loosening, a bone scan was performed. This type of imaging may not be a routine practice in all hospitals and may result in false-positive scans when performed within 2 years after implantation for hips and within 5 years for knees. However, because the negative predictive value is high, we considered it as a valuable diagnostic tool to rule out infection [30]. Finally, we only studied CRP and ESR as serum inflammatory markers. There are other sensitive serum markers available, such as D-dimer and interleukin 6 (IL-6) that we have not incorporated in our daily clinical care [31, 32]. Although these markers do not exhibit a diagnostic accuracy of 100% either, applying a combination of serum inflammatory markers may be beneficial [33, 34].

In conclusion, in case of prosthetic loosening, all joints need to be aspirated before revision surgery as no specific characteristic could be identified to 100% rule out an infection. A prosthetic implant that needs to be revised for a mechanical reason without any signs of loosening probably does not need a synovial fluid aspiration, in particular, not when combined with low serum inflammatory markers.
